# Immigration attitudes among Western and Eastern European MPs: social identity, economic aspects and political ideology

**DOI:** 10.1057/s41295-021-00254-5

**Published:** 2021-09-05

**Authors:** Bojana Kocijan, Marko Kukec

**Affiliations:** 1 Central European University, Budapest, 1051 Hungary; 2 Faculty of Economics and Social Sciences, Helmut Schmidt University, 22043 Hamburg, Germany

**Keywords:** Immigration, Members of parliament, Religion, Economy, Ideology, Survey

## Abstract

This article calls for greater attention to immigration attitudes of members of national parliaments (MPs) who absent harmonized immigration policy at the EU level remain the chief decision-makers and are thus responsible for swift government reaction to large influx of immigrants as witnessed in summer 2015 and spring 2020. Against this background, attitudes of MPs toward non-EU immigrants can be highly informative for understanding the foundation and direction of future immigration policy reforms. Although knowledge of MPs immigration attitudes is seemingly important, studies interested in this topic remain scarce. To test the relative importance of identity and economic aspects of MPs' immigration attitudes, this study adopts few well-established theoretical approaches from citizen-level research. Our data come from an MP survey that was administered in 11 Western and Eastern European countries in late 2014 as part of the *European National Elites and the Crisis* project. Our results suggest that social identity (religiosity) along with political ideology rather than economic concerns drive MPs' immigration attitudes. In addition, we find that in Eastern Europe immigration is only a light force behind political competition unlike in Western Europe, while economic left in Eastern Europe is more anti-immigrant than in Western Europe.

**Supplementary Information:**

The online version contains supplementary material available at 10.1057/s41295-021-00254-5.

## Introduction

Since the end of the Second World War migration to Europe unfolded in several waves. A wider geopolitical event such as 2003 Iraq conflict or Arab Spring in 2011 triggered waves distinct in immigrant populations. The most recent arrivals subsequent to Syrian crisis in 2015 and 2020 were the most diversified in terms of country of origin, migration motives and structure of migrant populations (Arango 2012; Van Mol and de Valk 2016). Historical migratory waves document that immigration is not an unusual or insurmountable challenge for host societies. However, large numbers of Muslim immigrants along the European Union (EU's) border in summer 2015 and in spring 2020 clearly show that immigration may become a potent socioeconomic and political challenge for host countries where prompt and adequate government reactions are called for. In summer 2015, Germany welcomed over a million of Middle Eastern immigrants (Tausch 2016), while Hungary built a fence on its borders with Serbia and Croatia to contain illegal immigration (Simonovits 2020).

Absent harmonized EU immigration policy these contrasting approaches to immigration by EU members call for greater attention to immigration attitudes of national elites. Immigration attitudes are commonly studied at citizen level while elite attitudes across Europe are widely neglected (Davidov et al. 2020; Hainmueller and Hopkins 2014). To fill this gap, we take on a rare effort to explore *immigration attitudes among national parliamentary elite (MPs) across Western and Eastern EU member states.* MPs are top-ranking politicians with legislative expertise, the ability to influence policy-making and wide powers to control the government (Yamamoto 2007). As experts, they may influence positions of their parties on immigration and participate in various EU immigration focus groups (Oliveira et al. 2014). The study of immigration attitudes is an important complement to a better established manifesto-based research because analyzing individual MPs can account for heterogeneity of immigration preferences within a single party (Odmalm and Super 2014b). It also has important implications for political representation, policy-making and political polarization. Different considerations MPs take in the account when thinking about immigration might affect the agenda of political competition or intensify polarization where it was previously low or moderate. Also, the way MPs see immigration may influence citizens’ opinion, thus forming and/or strengthening political representation (Magnani 2012).

To explain immigration attitudes of MPs, we use social identity theory, economic threat theory and political ideology. Citizen-level studies yield support for cultural concerns as well as political partisanship in explaining immigration attitudes, while personal economic circumstances have less explanatory power (Hainmueller and Hopkins 2014). Furthermore, our work is a pioneering study of elite-level immigration attitudes across Western and Eastern Europe. We not only consider different levels of immigration across the two regions, but also look at how different past experiences, economic and demographic contexts influence the relative importance of cultural compared to economic considerations in terms of immigration attitudes. Comparing these two regions is warranted because countries within these regions share similar socioeconomic characteristics, but are still profoundly different from one another. While Western EU countries are established democracies with robust economies and high levels of immigration, Eastern EU countries share a communist past, weaker degree of economic development and low levels of immigration.

Given our interest in elite-level immigration attitudes across Western and Eastern Europe we rely on the data collected in late 2014 as part of the *European National Elites and the Crisis* (ENEC) project that administered a survey among MPs in eleven EU member states: Bulgaria, Croatia, France, Germany, Greece, Hungary, Italy, Lithuania, Portugal, Slovenia and Spain. In line with the citizen-level literature our data record that social identity (religiosity) and political ideology (positions on general left–right scale) rather than economic prospects influence immigration attitudes of national MPs. Furthermore, we find that immigration did not yet fully enter political competition in Eastern Europe. Eastern European MPs positioned further to the right of the ideological scale are not more anti-immigrant than Western European MPs. On the other hand, economic left in Eastern Europe tends to be more anti-immigrant than economic left in Western Europe.

We first review citizen-level literature on immigration attitudes and develop our hypotheses. We then present our data, ordered logistic models and results. In the conclusion, we discuss implications of our findings for future research and address study’s shortcomings.

## Sources of immigration attitudes

### Economic threat theory

According to economic threat theory immigration is the source of labor market competition between natives and immigrants for jobs or government services (Esses et al. 1998; Gorodzeisky 2011; Quillian 1995). The citizen-level literature probed many economic aspects looking for a relationship to immigration attitudes. Immigrants may be willing to take low-paid jobs thus triggering exclusionist attitudes. However, professional skills of immigrants may mediate immigration attitudes. High-skilled natives may prefer low-skilled immigrants who are willing to take jobs that do not appeal to the natives (Mayda 2006; Scheve and Slaughter 2001). Similarly, low-skilled immigrants may be a burden on public finances, whereas high-skilled immigrants have the opposite effect (Hanson et al. 2007).

The association between *national economic prospects* and immigration attitudes has also been documented by scholars (Sides and Citrin 2007), who argue that threat perception not only depends on one’s own individual economic situation but on that of one’s group (Meuleman et al. 2020). MPs are high-ranking officials guaranteed stable employment that comes with lucrative benefits and are generally better educated than populations at large (Best and Cotta 2000). Thus, we turn to *sociotropic estimates of group rather than individual economic prospects* (Hainmueller and Hopkins 2014; Hjerm 2009). In times of economic growth, low unemployment and positive economic prospects, natives may be more hospitable to immigrants (Wallace and Figueroa 2012). In addition, as experts with advanced knowledge of national and EU economies MPs have a better understanding of market needs for immigrant labor and fiscal impact of immigration on interdependent EU economy.

#### H1:

*MPs more pessimistic about the future state of the EU economy hold stronger anti-immigration attitudes compared to more optimistic MPs.*

Western and Southern European countries are ‘classical immigration countries’ (Okólski 2012), while Eastern European countries are the main source of emigration to Western Europe, especially subsequent to EU enlargements in 2004 (Favell 2008). Eastern European economies are comparatively much weaker than Western European economies, recording lower levels of economic growth and higher unemployment rates (Barysch 2006; Mansoor and Quillin 2006; Winiecki 2013). These countries are also beneficiaries rather than contributors of large EU financial transfers (Mattila 2006), which makes them heavily dependent on Western EU economies. Scholars also found evidence for higher levels of anti-Muslim prejudice amidst unemployment in Eastern Europe compared to Western Europe (Strabac and Listhaug 2008).

#### H2:

*MPs more pessimistic about the future state of the EU economy hold stronger anti-immigration attitudes compared to more optimistic MPs, but the effect is attenuated in Western Europe.*

### Religiosity

Religious identity is based on common beliefs, practices and the moral authority of religious teaching sustained over a long period of time (Ysseldyk et al. 2010: 61). According to social identity theory, religion is the source of group identification that may induce symbolic identity threat if religion of immigrants is dissimilar to that of natives (McDaniel et al. 2011; Scheepers et al. 2002). The *behavioral* dimension of religion understood as participation in religious services (*church attendance)* is especially important in formation of strong group attachments and religious social identities (Ben-Nun Bloom et al. 2015: 3).

In contrast to religious behavior best observed as church attendance, religious *beliefs* may motivate compassion toward those in need, which may in turn trigger positive attitudes toward immigrants (Wald and Wilcox 2006; Knoll 2009; Strømsnes 2008). However, the effect is contingent upon religion of immigrants because pious individuals may be more compassionate only toward immigrants from their own religious community (Ben-Nun Bloom et al. 2015; Ford and Mellon 2020; Knoll 2009). As Christianity^1^ is an important source of cultural and religious European heritage and immigrants are an out-group strongly associated with Islam (Casanova 2007; Storm 2011: 75), we expect religiosity would trigger negative immigration attitudes:

#### H3:

*The more religious MPs are, the stronger are their anti-immigration attitudes.*

Although the literature repeatedly found that Eastern Europeans are more attached to their cultures (Ceobanu and Escandell 2010) and hold stronger anti-immigrant attitudes than Western Europeans (Davidov et al. 2020), less attention was hitherto paid to the relationship between religiosity and immigration attitudes across the two European regions. Following a spectacular revival of religiosity in Eastern Europe in 1990s, both Western and Eastern Europe have experienced a steady and continuous secularization in past decades (Apahideanu 2013). However, Eastern European Catholic and Orthodox churches are sources of cultural and national identities and informal institutions that stand up against ‘external forces’ such as for example communism in the past and more recently globalization (Coutinho 2016). Consequently, Eastern European MPs closely attached to the church they perceive as protector of cultural identity might be wearier of Muslim immigrants than religious Western European MPs. Not surprisingly, scholars found that Eastern Europeans are generally more intolerant of Muslim immigrants than Western Europeans (Doebler 2014). Hence, we expect the effect of religiosity on anti-immigration attitudes to be positive for MPs from Eastern Europe, while it should be attenuated among Western European MPs.

#### H4:

*The more religious MPs are, the stronger are their anti-immigration attitudes, but the effect is attenuated among Western European MPs.*

### Political ideology

#### General left–right ideological placement

Political ideology expressed as general left–right placement has consistently proven to be a reliable predictor of citizens’ immigration attitudes (Hainmueller and Hopkins 2014; Sides and Citrin 2007). At the elite level, the effect of political ideology is even more relevant because it empowers the representational link between MPs and voters. The ideological placement of MPs should give voters cues about MPs' positions on immigration and other policy issues (de Vries et al. 2013: 223). Political ideology is a multidimensional concept composed of economic and value dimensions (Coman 2017) and both dimensions should be accounted for to properly understand the effect of left–right ideological placement on immigration attitudes. There is, however, ample evidence for cultural and value rather than economic orientations to be the primary components of general left–right ideological dimension in Europe (Coman 2017; Henjak 2010; Kriesi 2010). Therefore, we consider the general left–right placement of MPs to be an expression of their cultural and value orientations.

Attitudes toward ‘others’ and globalization are currently the most salient components of cultural political divide in both Western and Eastern Europe (de Vries et al. 2013; Hooghe and Marks 2018; Teney et al. 2014). In their citizen-level study on globalization and immigration attitudes Teney et al. (2014) show that ‘cosmopolitan’ and ‘communitarian’ views are the most powerful predictors of immigration attitudes among Europeans. On the one hand, ‘communitarians’ hold strong national attachments, which is a trait commonly associated with right-wing MPs and less hospitable immigration attitudes. On the other hand, ‘cosmopolitans’ hold loose national attachments and are more prone to opening national borders for international exchange, which is a trait commonly associated with left-wing MPs. Hence, we expect the relationship between MPs’ general left–right self-placement and their anti-immigration attitudes to be positive.

##### H5:

*MPs positioned further to the right of the general left–right ideological scale have stronger anti-immigration attitudes compared to MPs positioned further to the left of the general left–right ideological scale.*

The extent to which immigration divides MPs with left-wing and right-wing value orientations may depend on politicization of immigration in a respective society (Manevska and Achterberg 2011: 8). Immigration did not structure early post-war Western European party systems to a large extent. However, as immigration levels increased in recent decades it became more salient (Dennison 2019: 14) and did contribute to an increasing polarization between left-wing and right-wing MPs. The manifesto-based studies of polarization at the party level have largely confirmed this trend for Western Europe (Akkerman 2015; Alonso and Claro da Fonseca 2012). The present analysis of individual MPs’ immigration attitudes explores whether this realignment recorded in the West is merely an instrument of political competition or whether it was also internalized by political elites. In contrast to Western Europe, immigration to Eastern Europe in recent decades has remained relatively limited (Van Mol and de Valk 2016: 64–65). This in turn kept the immigration issue outside of political discourse and consequently resulted in blurred dividing lines between left and right. The analysis of party manifestos confirmed that mainstream parties in Eastern Europe failed to react to the rise of radical right parties and did not position themselves more clearly on issues such as nationalism and immigration (Heinisch et al. 2019: 10). Empirical studies at the citizen-level found a lower effect of left–right value orientation on immigration attitudes in Eastern Europe (Ceobanu and Escandell 2010: 321; Citrin and Sides 2008: 47–48), which gives us confidence to expect the same conditional effect for Eastern European MPs.

##### H6:

*MPs positioned further to the right of the general left–right ideological scale have stronger anti-immigration attitudes compared to MPs positioned further to the left of the general left–right ideological scale, but the effect is attenuated among Eastern European MPs.*

#### Economic left–right ideological preference

Economic dimension of ideology may also shape MPs’ immigration attitudes. Conflict over economic policy between left and right revolves around involvement of the state in the economy, particularly issues of redistribution, labor market regulation and public ownership. It traditionally pitted left-wing favoring greater involvement of the state against market-oriented right-wing. Immigration maps onto this ideological dimension. Particularly, immigration may lower wages of blue-collar workers, undermine collective bargaining and increase pressure on the welfare state (Esses et al. 1998). To protect their traditional working class constituency against these pressures economic left wing might be more prone to adopt skeptical attitudes toward immigration. In contrast, advocates of market mechanism may favor immigration as it increases competitiveness of the economy for its provision of inexpensive labor (Odmalm and Super 2014a: 304).

Some authors pointed to the immigration dilemma by socialist and social democratic parties (Carvalho and Ruedin 2018; Odmalm and Super 2014a). On the one hand, left-wing parties traditionally advocate cosmopolitanism, solidarity and liberal sociocultural values, which triggers positive sentiment toward immigration. On the other hand, their economic concerns for working class may lead left-wing parties to adopt more skeptical immigration attitudes. Focusing on economic preferences of individual MPs while controlling for their value orientations allows us to explore more precisely the effect of left-wing economic preferences on immigration attitudes.

##### H7:

*MPs who prefer social security over economic competitiveness hold stronger anti-immigration attitudes.*

A restricted inflow of immigrants to Eastern Europe should ease concerns of economic left over pressure immigrants pose to domestic labor market and welfare state. Moreover, *emigration* from Eastern Europe resulted in some marginal improvements of labor market conditions contributing to increase in wages and decline in unemployment (Kahanec 2015: 367). Workers from neighboring states, including Belarus, Moldova, Russia and Ukraine, usually make up a small percentage of work force sufficient to replenish labor market shortages incurred by emigration (Gödri and Csányi 2020: 505; Mikalauskiene et al. 2017: 38). Hence, in Eastern Europe economic conditions for politicization of labor immigration are relatively scarce. This makes us confident to expect that economic left in Eastern Europe is less likely to hold anti-immigration attitudes compared to economic left in Western Europe.

##### H8:

*MPs who prefer social security over economic competitiveness hold stronger anti-immigration attitudes, but the effect is attenuated among Eastern European MPs.*

## Data, variables and model

Empirical analysis is based on the MP survey carried out in late 2014 in eleven Western and Eastern EU member states (Bulgaria, Croatia, France, Germany, Greece, Hungary, Italy, Lithuania, Portugal, Slovenia and Spain). Country teams used quota sampling to secure balance across all relevant MP groups, including gender, political experience and party affiliation. A total of 717 MPs responded to the standardized questionnaire (see online appendix).

We measure (*anti)immigration attitudes* with the following survey question: *Do you think that immigration from non-EU countries is a threat or not a threat to the EU*? The resulting variable is ordinal with four categories: ‘not at all a threat’ (1), ‘not that big of a threat’ (2), ‘quite a big threat’ (3) and ‘a big threat’ (4). Given the pressures of globalization on domestic labor markets and welfare state as well as large numbers of immigrants from Middle East and North Africa (MENA) (Greenhill 2016: 323) prior to the year of our survey, we are confident that our measure captures both cultural and economic aspects of immigration.

Independent variable that measures *sociotropic economic evaluation* is based on the following survey question: ‘*Thinking about the future of the EU, we would like to know what do you think will happen in 10 years – the economy of the EU as a whole will be (1) more robust, (2) less robust, or (3) there will be no significant change.’* Absent a more appropriate measure we rely on MPs’ perspectives on the future of the entire EU economy. MPs’ perspectives about the overall EU economy should nevertheless reflect MPs’ perspectives of their national economies given that EU economies are strongly integrated. Particularly, MPs from ‘net beneficiary’ member states might be especially worried about potential spillover effect in the event of EU-wide economic decline.

We measure behavioral aspect of MPs’ *religiosity* as religious attendance: ‘*About how often do you attend religious services?*’ (1–5 scale). A separate question about MPs’ religious belonging reveals that about two-thirds of MPs in our sample are Christian, one-third agnostic or atheist and only 1% Jewish or Muslim. Therefore, our measure of religious attendance rather precisely reflects a degree of attachment of MPs to Christian faith. A disadvantage of our church attendance measure is that 121 out of 717 MPs refused to report how frequently they attend religious services. As a robustness check, we rerun our models using an alternative measure of cultural identity, namely opinions of MPs about the extent to which the EU endangers their national cultures.

We use standard 11-point general left–right scale to measure *value component of MPs’ political ideology*. *Economic component of political ideology* is measured as an agreement of MPs with one or both of the following statements: (1) ‘*The main aim of the EU should be to make the European economy more competitive in world markets*’ (2) ‘*The main aim of the EU should be to provide better social security for all its citizens.*’ We use a binary variable to discriminate between West and East and interact it with four independent variables to test their differentiated effect across the two European regions. All models control for MPs’ *age*, *gender* and *education*.

Since our dependent variable has four ordered response categories we use ordered logistic regression with standard error corrected for country clustering of MPs. Our coefficients are expressed as odds ratios to indicate the odds of falling into a certain category against the odds of falling into all lower categories combined. The test for parallel regression assumption suggests that ordinal logistic regression is a valid procedure. We report descriptive statistics of our variables in Table 1.

**Table 1:** Descriptive statistics of dependent and independent variables

Variable	N	Mean/Percentage
Immigration as a threat	701	
Not at all	190	27.1%
Not big	237	33.81%
Quite big	175	24.96%
Big	99	14.12%
EU economy in 10 years	674	
More robust	458	67.95%
Less robust	150	22.26%
No significant change	66	9.79%
Religiosity	596	3.08
General left–right position	682	4.79
Economic policy	698	
Competitiveness	221	31.66%
Social security	283	40.54%
Both	194	27.79%
East	717	39.61%
Age	711	49.59
Gender (female)	210	29.29%
Education (university)	713	88.08%

## Results

Table 2 presents the results of five multivariate ordered logistic regression models. Model 1 includes the average effects of our independent variables, while subsequent models (2–5) test their heterogeneity across Eastern and Western Europe. The effects of control variables are non-significant across all models. The results of Model 1 do not support the argument that sociotropic evaluations of the EU economy influence MPs’ (anti)immigration attitudes. Contrary to our expectation in H1, MPs who think the EU economy will be ‘more robust’ in the next ten years do not hold significantly different immigration attitudes compared to MPs who think the EU economy will be ‘less robust.’ In contrast, the effect of frequency of religious attendance is statistically significant and positive, which corresponds to H3. The effect is moderately substantial as MPs who never attend religious services have about 8% predicted probability of falling into the highest category of immigration threat perception, while regular religious attendance increases the predicted probability to 18%.

**Table 2:** Ordered logistic regression output

Threat of non-EU immigration	Model 1	Model 2	Model 3	Model 4	Model 5
EU economy^a^					
*Less robust*	1.15 (0.27)	1.06 (0.38)	1.26 (0.31)	1.43 (0.42)	1.32 (0.36)
*No change*	2.14 (0.13)	1.50 (0.71)	1.64 (0.58)	1.56 (0.53)	1.53 (0.45)
EU economy^a^					
Less robust*East	–	1.64 (0.93)	–	–	–
No change*East	–	1.16 (0.72)	–	–	–
Religiosity	1.27* (0.13)	1.27* (0.12)	1.34* (0.19)	1.25* (0.10)	1.28* (0.12)
Religiosity*East	–		0.88 (0.17)	–	–
Left–right	1.30** (0.11)	1.30** (0.11)	1.31** (0.11)	1.60*** (0.10)	1.31** (0.11)
Left–right*East	–	–	–	0.73** (0.09)	–
Economic orientation^b^					
*Social security*	0.78 (0.19)	1.02 (0.28)	1.02 (0.28)	1.09 (0.28)	0.48 (0.27)
*Both*	0.73 (0.18)	0.94 (0.23)	0.95 (0.23)	0.92 (0.23)	0.52 (0.26)
Economic orientation^b^					
Social security*East	–	–	–	–	4.04* (2.44)
Both*East	–	–	–	–	2.74* (1.36)
East	–	2.42** (0.70)	3.95* (1.93)	14.59*** (10.34)	1.22 (0.69)
Age	1.01 (0.11)	1.01 (0.01)	1.01 (0.01)	1.01 (0.01)	1.01 (0.01)
Female	0.74 (0.15)	0.73 (0.15)	0.71 (0.16)	0.72 (0.17)	0.75 (0.15)
University education	1.52 (0.48)	1.50 (0.42)	1.44 (0.42)	1.45 (0.44)	1.45 (0.42)
Observations	514	514	514	514	514
Log likelihood	− 634.13	− 618.84	− 618.86	− 609.21	− 613.39
Pseudo *R*^2^	0.09	0.11	0.11	0.12	0.12

Table entries are odd ratios; cluster-corrected standard errors in parentheses

*p < 0.05, **p < 0.01, ***p < 0.001

^a^Reference category: More robust

^b^Reference category: Competitiveness

The relative importance of identity is also reflected in the average effects of ideological orientations. MPs who position themselves further to the right of the general left–right ideological scale have significantly stronger anti-immigration attitudes, which corresponds to H5. The effect is substantial, as extreme-right MPs have 30 percentage points higher odds of falling into the highest category of threat perception compared to extreme-left MPs. The average effect of preference for social security over market competitiveness is not statistically significant, which is contrary to H7. These results provide external validation of our assumption that general left–right ideological scale mainly captures value and identity orientations of MPs as its effect is substantial and corresponds to expected direction, while the separate effect of economic ideological orientation is not statistically significant.

**Fig. 1:**
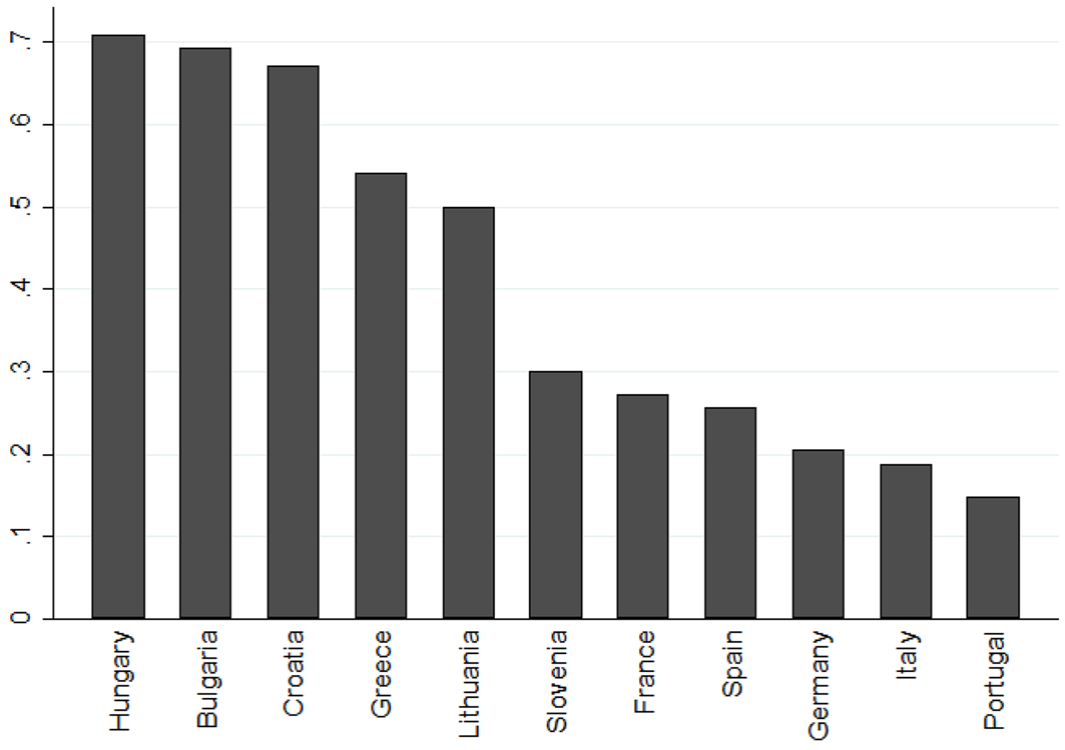
Anti-immigration attitudes among MPs in eleven EU countries

Models 2 and 3 test the heterogeneous effect of economic perception and religiosity across Eastern and Western EU by interacting these variables with a binary variable indicating MPs from Eastern Europe. As reported in Fig. 1, there is a stronger prevalence of anti-immigration attitudes among Eastern European MPs. However, the effect of economic perception and religiosity does not differ across the two European regions. Hence, empirical analysis does not confirm H2 and H4.

**Fig. 2:**
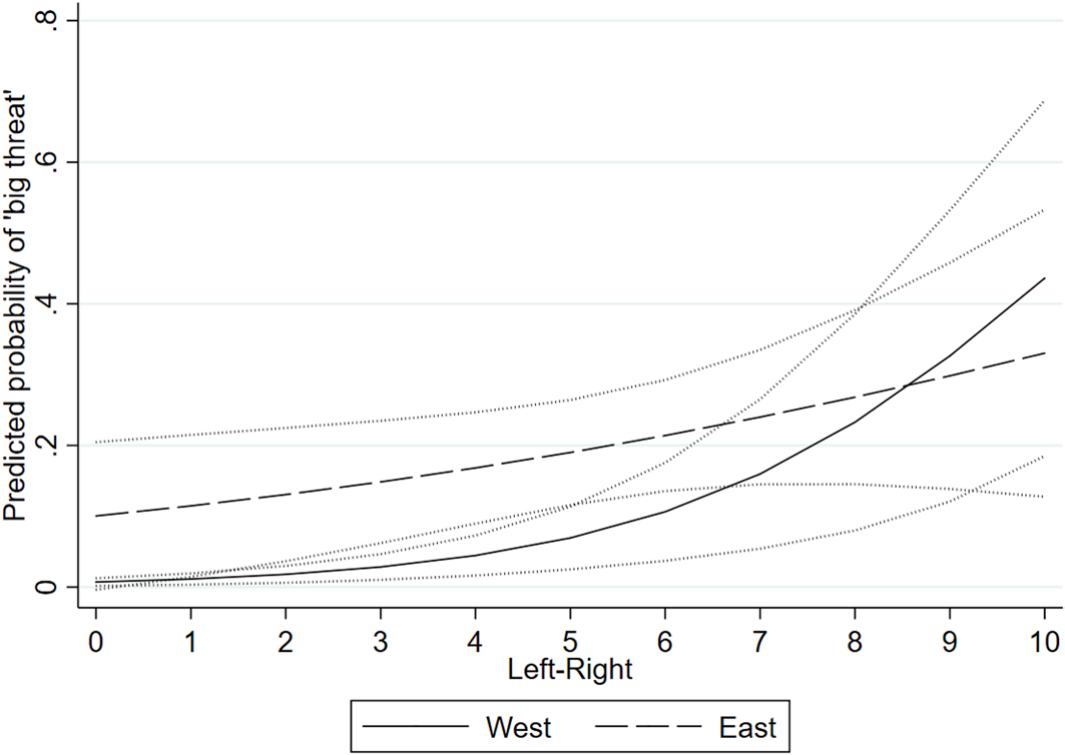
The effect of general left–right ideological placement in Western and Eastern Europe

The effect of ideological orientations does, however, vary across the two regions. The results of Model 4 report a statistically significant negative interaction term for the general left–right ideological scale, which implies a weaker effect in Eastern Europe. To visualize the interaction effect Fig. 2 plots predicted probabilities of falling into the ‘big threat’ category. Extreme-left MPs from Western Europe have almost zero likelihood of falling into the ‘big threat’ category. However, as we move toward the right end of the spectrum the curve grows steeply with extreme-right MPs reaching 44% likelihood of falling into the highest category of threat perception. In Eastern Europe, the effect of general left–right ideological positioning is positive, but the slope indicates a rather weak (and non-significant) relationship to immigration attitudes.

**Fig. 3:**
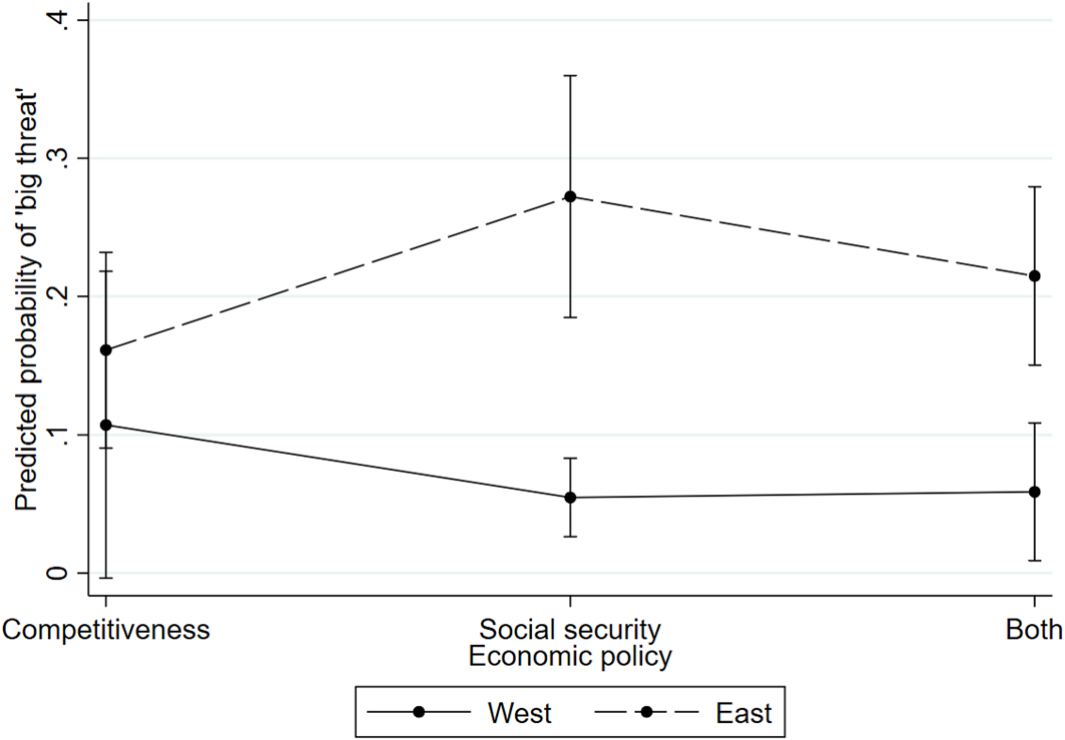
The effect of preference for social security over competitiveness in Western and Eastern Europe

As the average effect of economic ideological orientations is rather weak, it is quite surprising that the effect of preference for social security is significantly higher in Eastern Europe. Expectations in H8 would rather imply that if there was any effect of this variable it should apply to MPs in Western rather than in Eastern Europe. Fig. 3 illustrates substantively higher anti-immigration attitudes of Eastern European MPs who prefer social security compared to those who prefer competitive economy. Eastern European MPs who prefer a middle way between competitiveness and social security are also moderate in their immigration attitudes, which additionally validates the pattern found in Eastern Europe.

In the final step of the empirical analysis, we test the robustness of our results to the exclusion of MPs who refused to report the frequency of their religious attendance. We do this because first, MPs who did not report their religious attendance might be substantially different from MPs who did, which would then bias our results that emphasize relevance of values and identity in reference to immigration. Second, MPs who did not report the frequency of their religious attendance might systematically differ on other characteristics relevant for this study. To perform the robustness test we replace ‘religiosity’ with ‘cultural threat’ variable which also taps into the identity aspect giving cues about MPs’ opinions whether the EU endangers their national cultures. The results reported in the online appendix (Table A1) confirm that the findings hold even on the larger unbiased sample of MPs. ‘Cultural threat’ variable is significant across all five models, but its effect does not vary across East and West, while the effect of other variables remains substantively similar across models.

## Discussion

Our analysis yields several notable findings related to the relative importance of identity over economic concerns, as well as the heterogeneity of their effects on (anti)immigration attitudes across Eastern and Western Europe. The analysis broadly confirms that compared to economic concerns, social identity and value orientations bare more relevance in explaining MPs’ immigration attitudes. This finding complements previous citizen-level studies which repeatedly confirm cultural variables to more successfully explain hostility toward immigrants than economic (rational) calculations (Ben-Nun Bloom et al. 2015; Sides and Citrin 2007).

Our analysis further confirms that despite secularization and decreasing participation in religious events, Christianity as an integral part of European identity still plays an important role in shaping attitudes toward out-groups (Storm 2011: 76). Over the last two decades, influx of Muslim immigrants and increasing attention paid to immigration sustains religion as the main cultural demarcation line between Europeans and immigrants. Large arrival of mostly Muslim immigrants seemingly triggers stronger anti-immigrant attitudes raising concerns of religious MPs about preservation of Christianity as the main aspect of European identity. The self-placement of MPs closer to the right pole of the general left–right ideological scale, which is strongly associated with ‘communitarian’ values has a substantive positive effect on anti-immigration attitudes. This finding further confirms the relevance of identity and value considerations in formation of immigration attitudes.

It is somewhat surprising that MPs generally do not base their immigration attitudes on rational economic considerations. As members of parliamentary elite with advanced knowledge about costs and benefits of immigration, as well as the material capacities of their societies to support immigrants, MPs should be in a position to evaluate the impact of immigration on labor market and economic growth. However, neither the evaluation of the future strength of the EU economy, nor MPs’ preference for a greater or lesser involvement of the state in the economy explain their (anti)immigration attitudes.

Our study is in line with Matonyte and Morkevičius (2012) who found that Eastern European MPs on average consider immigration a greater threat than Western European MPs. Although some scholars found immigration attitudes to be converging between East and West subsequent to EU enlargements in 2004 (Ceobanu and Escandell 2010) our data show that at the elite-level differences remain. This finding challenges the argument that higher proportion of immigrants drives exclusionist immigration attitudes. Higher prevalence of anti-immigration attitudes in Eastern Europe with fewer immigration populations compared to Western Europe is likely related to communist legacies, lower levels of tolerance and weaker democratic traditions coupled with precarious economic transition and weaker economic standards (Kunovich 2004; Strabac and Listhaug 2008). Moreover, intergroup contact theory argues that frequent contacts between different groups might actually reduce unfavorable out-group attitudes (Schlueter and Scheepers 2010: 287). However, since immigrant populations in Eastern Europe are rather small, the likelihood of such positive contacts is low.

The effects of MPs’ religiosity and perspective on the economy do not vary across Eastern and Western Europe. Religiosity remains an important component of Western European identity, despite the purported secularization of these societies (Storm 2011). In Eastern Europe, religion witnessed an incredible upsurge in early 1990s as the major force behind political changes especially in Catholic countries. Hence, the role of religious identity as the basis for MPs’ immigration attitudes does not differ between the two regions. The absence of cross-regional heterogeneity of the effect of sociotropic economic concerns on immigration attitudes can less credibly be rationalized on the basis of the extant literature. We need more research on both citizen and elite level to shed light on this relationship.

The effect of MPs’ ideological orientations seems to be even more context-dependent. We suspect this to be related to constructed and malleable nature of ideological orientations, which is known to vary based on societal structure, historical circumstances and politicization of particular issues (Coman 2017). The effect of general left–right ideological orientation is particularly pronounced in Western Europe where continuous immigration over the last half-century placed immigration at the center of political debate forcing ‘communitarians’ and ‘cosmopolitans’ to take a clear stance on its relative merits. The effect in Eastern Europe is considerably lower, which could be traced back to lower size of immigrant community and the resulting lower politicization of the issue.

The effect of ideological orientations related to the involvement of the state in the economy is likewise context-dependent, but not as expected. While the issue salience argument would expect immigration to divide economic left and economic right particularly in Western Europe because of its large immigrant populations we found this effect only in Eastern Europe. Without a more thorough analysis which exceeds the scope of the present study we may only speculate about the reasons for this counterintuitive effect. It could be that the continuing instability of the economy in Eastern Europe was particularly exposed during the most recent economic crisis. In Eastern European countries covered by the analysis GDP fell considerably and unemployment dominated the national agenda throughout the crisis (Fink-Hafner 2013: 219; Kolarova and Spirova 2013: 38; Krupavicius 2012: 189). This economic hardship could explain why economic left in Eastern Europe tends to assume more exclusionary attitudes toward immigration that would inevitably place additional pressure on domestic workforce and welfare state.

## Conclusion

This study offers a rare comparative insight into the immigration attitudes of MPs. This under-researched topic bares relevance since MPs are the main national decision-makers able to influence public opinion, and their parties’ policy positions on various issues including immigration. The analysis of MPs from eleven Western and Eastern European countries reveals three notable patterns. First, identity and cultural aspects more powerfully shape MPs’ immigration attitudes compared to their economic orientations. The substantive effects of religiosity and general left–right ideological position demonstrate the preference of pious and identity-oriented right-wing MPs for in-group symbolic closure. Second, the importance of economic orientations should not be completely disregarded, as particularly Eastern European MPs who prefer redistribution over market competition are more concerned about immigration. Although the economic crisis might have raised concerns of Eastern European economic left over domestic labor market and sustainability of welfare states, more focused case studies should explore the precise mechanism behind this effect. Third, the effect of general left–right ideological orientation is stronger in Western Europe, which implies that immigration is a politically more salient and divisive issue among Western European MPs, but has no baring among Eastern European MPs.

Our findings of relative importance of cultural versus economic aspects related to MP’s immigration attitudes contributes to better understanding of the key roles they play as national decision-makers in immigration policy. Speaking to the literature on the origin of immigration attitudes at the elite-level (Dancygier and Margalit 2020; Helbling 2017; Magnani 2012), we show that cultural and symbolic compared to economic considerations play a more important role in formation of immigration attitudes. Previously, this pattern was confirmed for political parties, but less was known about the origin of immigration attitudes among individual representatives.

The fact that elected politicians base their immigration attitudes on cultural and symbolic considerations has important implications for political representation. On the one hand, comparative studies of immigration attitudes of the general population have likewise confirmed the prevalence of cultural considerations (Manevska and Achterberg 2011: 445). This testifies to the general citizen-MP congruence on how immigration should be approached. On the other hand, such congruence signals that political representation related to immigration is driven more by symbolic concerns rather than rational argumentation of economic costs and benefits. This affective (emotional) representation linkage could be further reinforced bearing in mind that citizens take cues from political elites when forming personal attitudes on complex and multidimensional issues such as immigration (Hellwig and Kweon 2016). Additionally, the emotional basis of elite immigration attitudes might stimulate polarization, particularly by those who feel that immigration threatens fundamental symbols of European culture and identity.

The emotional and symbolic nature of immigration debate may also implicate policy processes and outcomes. First, when emotions, symbols and identity are involved it might be more difficult for political elites to find compromise on immigration policy. Second, if MPs fall back on symbols when debating immigration, they might be less willing to react to changing economic circumstances (Brader et al. 2008).

Our analysis provides preliminary cues about the direction and basis for different policy measures such as immigrant quotas and integration programs that took place subsequent to most recent immigration arrivals amidst non-harmonized EU immigration policy. For example, our findings reflect the nature of national governments’ response to 2015 migration crisis. The decision to reimpose internal border controls or in German case welcome immigrants was grounded in perceptions of European leaders about immigration as a challenge to religious, cultural and linguistic foundations of European societies but also a potential to fill up labor shortages (Greenhill 2016).

There are several methodological limitations of our analysis which could be addressed in future work. Our dependent variable does not discriminate between ethnicity of immigrants. However, we believe that when asked about ‘non-EU immigration’ MPs widely thought of Muslim immigrants from MENA (Greenhill 2016: 323). The extant literature shows that people are less hostile to immigrants who culturally resemble them, so further research should in more detail address this mechanism at the elite level (Berg 2015; Ceobanu and Escandell 2010; Kentmen-Cin and Erisen 2017). In addition, larger number of countries or information about MPs’ constituencies would allow researchers to test different country- or constituency-level effects on MPs’ immigration attitudes (e.g. size of the immigrant population and/or economic wealth).

As we carried out our MP survey several months prior to the peak of 2015 migration crisis, our analysis does not fully capture its context. Hence, our study can be a solid baseline for future attempts to track the attitudinal consequences of increasing immigration, which is continuously triggered by political instability and poverty in European neighborhood. For example, Hooghe and Marks point to increasing salience of immigration among political parties in Eastern Europe subsequent to 2015 migration crisis, especially after the European Commission proposed to distribute asylum-seekers across the EU (2018: 126). Moreover, the origin of most recent immigration waves with predominately Muslim population from MENA might have reinforced the importance of MPs’ cultural aspects as a driving force behind their immigration attitudes in both Eastern and Western Europe. Finally, it would be interesting to explore whether symbolic aspects prevail over economic considerations in formation of MPs’ immigration attitudes once immigration crisis coincides with crisis of other natures such as recent Covid-19 health crisis, natural disasters (floods or earthquakes) and economic crisis.

## Supplementary Information

Below is the link to the electronic supplementary material.

Supplementary file1 (DOCX 34 KB)

## Footnotes

1 We acknowledge differences across Christian denominations (Catholics, Orthodox Christians or Protestants) could influence immigration attitudes based on distinctions in doctrinal teachings (Bohman and Hjerm, 2014). However, because of their acceptance of the same divinity as higher spiritual authority (Jacobsen, 2011), we are confident that Christian denominations all claim in-group membership in Christianity compared to out-group Islam.
