# Supplementary material for: Immigration attitudes among Western and Eastern European MPs: social identity, economic aspects and political ideology
Source: Comp Eur Polit. 2021 Sep 5;20(1):33–52. doi: 10.1057/s41295-021-00254-5 (PMC8418682; doi:10.1057/s41295-021-00254-5)
Supplement: Supplementary file 1 — Supplementary file1 (DOCX 34 KB) [file 41295_2021_254_MOESM1_ESM.docx]

# Online Appendix

*Table A1: Ordered logistic regression output (robustness check)*

| Threat of non-EU immigration | Model 1 | Model 2 | Model 3 | Model 4 | Model 5 |
| --- | --- | --- | --- | --- | --- |
| EU economy^1^ |  |  |  |  |  |
| *Less robust* | 1.00 (0.30) | 0.96 (0.38) | 1.12 (0.33) | 1.21 (0.39) | 1.20 (0.37) |
| *No change* | 1.65 (0.83) | 0.99 (0.31) | 1.23 (0.48) | 1.20 (0.43) | 1.15 (0.38) |
| EU economy^1^ |  |  |  |  |  |
| *Less robust*East* | - | 1.58 (0.89) | - | - | - |
| *No change*East* | - | 1.41 (0.90) | - | - | - |
| Cultural threat | 1.48** (0.21) | 1.39* (0.18) | 1.28 (0.17) | 1.40** (0.18) | 1.37* (0.18) |
| Cultural threat*East | - | - | 1.21 (0.37) | - | - |
| Left-right | 1.38*** (0.09) | 1.37*** (0.10) | 1.36*** (0.10) | 1.61 (0.13) | 1.37*** (0.10) |
| Left-right*East | - | - | - | 0.75** (0.10) | - |
| Economic orientation^2^ |  |  |  |  |  |
| *Social security* | 0.66 (0.14) | 0.88 (0.21) | 0.87 (0.20) | 0.92 (0.21) | 0.43 (0.19) |
| *Both* | 0.82 (0.17) | 1.05 (0.22) | 1.06 (0.22) | 1.01 (0.20) | 0.61 (0.24) |
| Economic orientation^2^ |  |  |  |  |  |
| *Social security*East* | - | - | - | - | 4.43** (2.27) |
| *Both*East* | - | - | - | - | 2.42** (1.02) |
| East | - | 2.41** (0.73) | 2.00 (1.06) | 12.16** (9.75) | 1.24 (0.62) |
| Age | 1.01 (0.01) | 1.01 (0.01) | 1.01 (0.01) | 1.01 (0.01) | 1.01 (0.01) |
| Female | 0.69* (0.13) | 0.69* (0.12) | 0.69* (0.13) | 0.66* (0.13) | 0.69* (0.12) |
| University education | 1.56 (0.42) | 1.45 (0.35) | 1.41 (0.36) | 1.39 (0.35) | 1.42 (0.36) |
| Observations | 604 | 604 | 604 | 604 | 604 |
| Log likelihood | -723.94 | -707.43 | -707.66 | -697.51 | -700.73 |
| Pseudo *R*^2^ | 0.10 | 0.13 | 0.12 | 0.14 | 0.13 |
| Table entries are odd ratios; cluster-corrected standard errors in parentheses  * p < 0.05, ** p < 0.01, *** p < 0.001  ^1^ Reference category: More robust  ^2^ Reference category: Competitiveness, category ‘Both’ omitted | | | | | |
